# Stimulated Emission Depletion Spectroscopy of Color Centers in Hexagonal Boron Nitride

**DOI:** 10.1021/acsphotonics.0c01917

**Published:** 2021-04-07

**Authors:** Ralph Nicholas Edward Malein, Prince Khatri, Andrew J. Ramsay, Isaac J. Luxmoore

**Affiliations:** † College of Engineering, Mathematics and Physical Sciences, University of Exeter, Exeter EX4 4QF, United Kingdom; ‡ Hitachi Cambridge Laboratory, Hitachi Europe Ltd., Cambridge CB3 0HE, United Kingdom

**Keywords:** photonics, color centers, hexagonal boron nitride, 2D materials, stimulated emission depletion

## Abstract

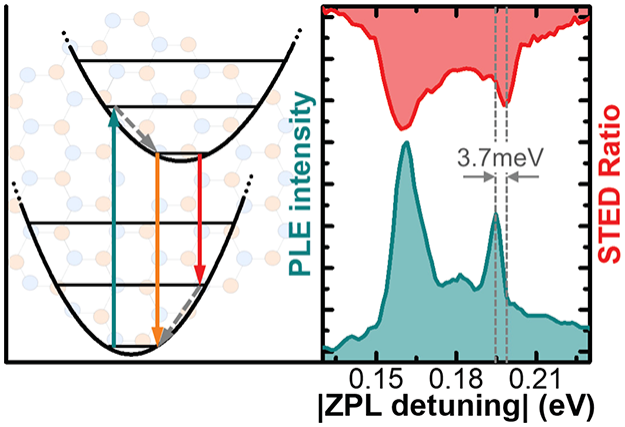

We demonstrate the use of Stimulated Emission Depletion (STED) spectroscopy to map the electron-optical-phonon sideband of the ground state of the radiative transition of color centers in hexagonal boron nitride emitting at 2.0–2.2 eV, with in-plane linear polarization. The measurements are compared to photoluminescence of excitation (PLE) spectra that maps the electron-optical-phonon sideband of the excited state. The main qualitative difference is a red-shift in the longitudinal optical phonon peak associated with *E*_1u_ symmetry at the zone center. We compare our results to theoretical work on different defect species in hBN and find they are consistent with a carbon-based defect.

The progress of photonic quantum technologies hinges on the development of components such as quantum light sources and memories. Due to their strong interaction with light and a wealth of fabrication and processing technology, atom-like solid state systems such as quantum dots or defects in wide-bandgap semiconductors hold much potential.^1,2^ One area of particular promise are light-emitting defects in hexagonal boron nitride (hBN), which show bright emission of single photons with narrow PL line widths compared to other single photon emitters in solids^3,4^ and Fourier transform-limited PLE line widths, even at room temperature,^5^ and with a low degree of emission into phonon-mediated modes.^6,7^ hBN’s graphene-like two-dimensional structure allows straightforward incorporation into photonic devices,^8−10^ and means that defects are always close to the device surface, which is desirable for the development of quantum sensors.^11,12^ Recently, optically detected magnetic resonance (ODMR) has been observed in hBN, establishing the possibility to host spin qubits.^13,14^

An open question in the field is the identity of the emitters. The issue is complicated by numerous candidate defects with similar zero-phonon line (ZPL) energies, motivating a search for additional spectroscopic signatures in ODMR,^13,14^ or in the phonon sidebands.^15−18^ For emitters at 2.0–2.2 eV, the strongest case to date has been made for a carbon-related defect.^19^

In this work we demonstrate stimulated emission depletion (STED) of color centers in hBN that emit around 2–2.2 eV. In STED an emitter is excited with two lasers with photon energies above and below the zero phonon line. The high energy laser pumps the emitter into the excited state, and the low energy laser stimulates phonon-assisted emission, thereby depleting the excited state and reducing the ZPL emission (Figure 1a). STED microscopy was developed for super-resolution spatial imaging^20−22^ and has also been used to perform subresolution limit photolithography,^23−25^ and to achieve lasing in NV centers in diamond.^26^ Here we apply STED as a spectroscopic probe, in combination with the complementary techniques of PL and PLE, to investigate the electron–phonon interaction of color centers in hBN. As illustrated in Figure 1a, STED and PLE probe the vibronic manifold of the radiative ground and excited states, respectively. In all emitters studied, we observe a phonon peak corresponding to Γ-point of longitudinal optical phonon of *E*_1u_ symmetry in bulk h-BN^27^ for both excited and ground electronic states. In the ground state, this peak appears 200 meV detuned from the ZPL. However, for the excited state this peak is red-shifted. From this we infer that the excited state induces a distortion of the lattice in the plane that is not present in the ground state. This distortion results in a small local shift to the *LO*(*E*_1u_) phonon mode energy.

**Figure 1:**
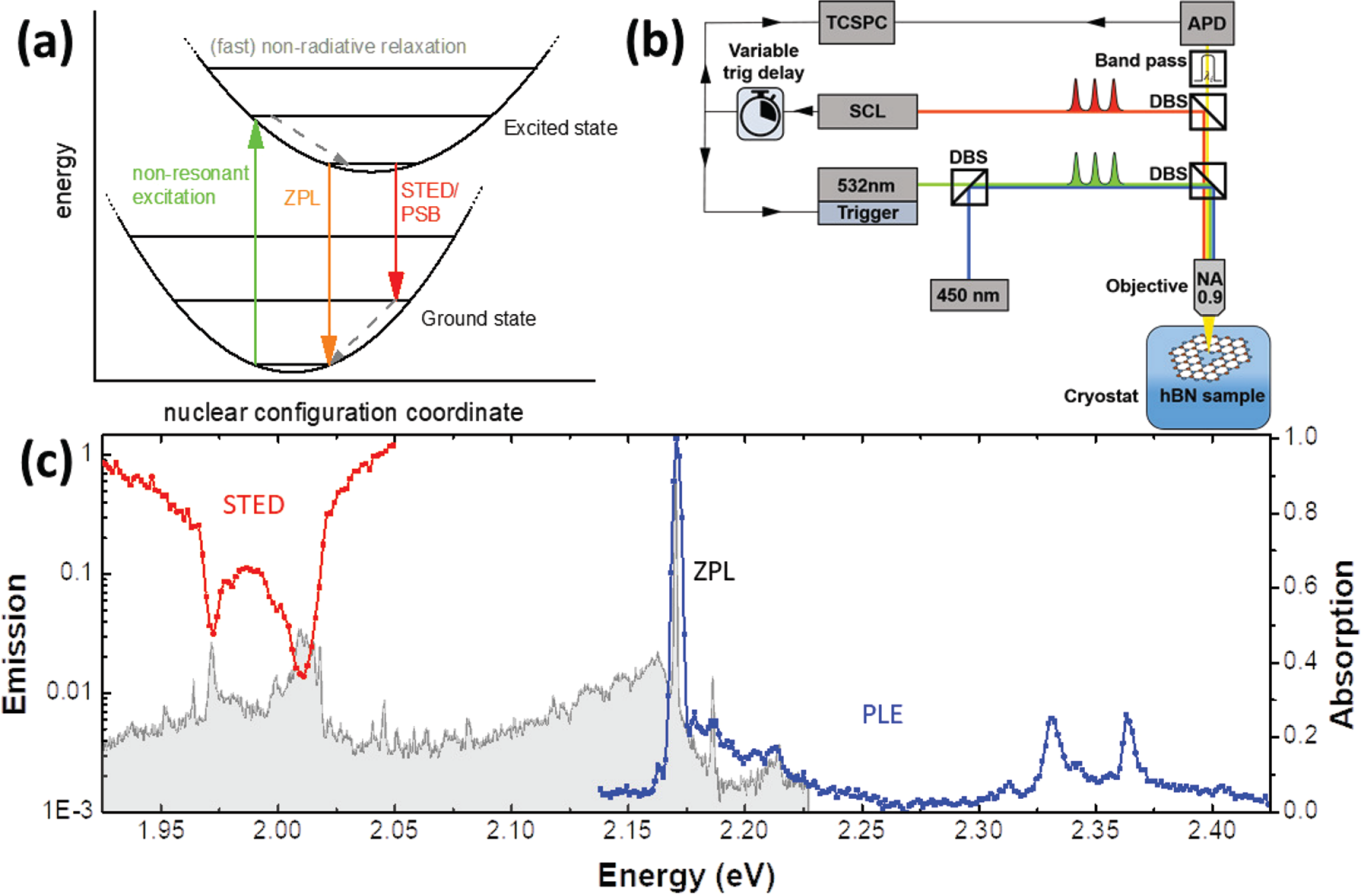
(a) Franck–Condon energy diagram comparing PL, PLE, and STED techniques. In PL, the nonresonant excitation (green) populates the excited state and emission from the ZPL (orange) and PSB (red) is collected. In PLE, the excitation probes the excited vibronic states and the ZPL is collected. In STED, the nonresonant excitation is again used, and the STED pulses (red) deplete the excited state through stimulated emission into the ground vibronic states, reducing the ZPL intensity. Gray dashed arrows show fast relaxation from higher vibronic states. (b) Diagram of the experimental setup. (c) Representative PL (gray), PLE (blue), and STED (red) spectra from defect-A with ZPL at ∼2.17 eV, emission OPSB between 1.97 and 2.03 eV, and absorption OSPB between 2.32 and 2.38 eV.

Figure 1a illustrates the principle behind STED by comparison to photoluminescence excitation (PLE). In PLE, the system is driven by a laser at a higher energy than the ZPL, and the ZPL is collected. By tuning the excitation laser, the vibronic spectrum of the excited state can be measured. Conversely, in STED, the system is pumped into the excited state, in this case, via nonresonant excitation, as in photoluminescence (PL), and again, the ZPL is collected. However, the system is then probed using a laser at a lower energy than the ZPL (red arrow). As this probe laser is tuned, stimulated emission via phonon-assisted transitions depletes the population of the excited state, reducing the intensity of the ZPL. The PL signal against probe laser energy shows the vibronic spectrum of the ground state, where dips in the PL signal correspond to phonon resonances. The STED spectrum replicates the PL spectrum, but as STED resonantly probes a specific transition, the spectral selectivity reduces extraneous signals and peaks from adjacent defects or impurities. As such, STED is useful for PSB measurements of emitters with random placement, and in "dirty" systems such as two-dimensional materials where defects, impurities, and surface effects are difficult to completely eliminate.

The samples examined consist of few-layer flakes of hBN dropcast from solution onto a Si substrate coated with a 5 nm layer of Al_2_O_3_.^28^ The flakes are annealed at 850 °C for 15 min in a N_2_ atmosphere to stimulate defect formation.^7^ Samples are then mounted in a closed-cycle cryostat and kept at 5 K. Figure 1b shows a schematic of the experimental setup. The lasers used for excitation and depletion are collimated and coaligned and then coupled to a long working distance objective lens, with numerical aperture of 0.8, which focuses the light to a diffraction-limited spot <1 μm in diameter. Light emitted from the samples is collected into the same objective and coupled into a spectrometer and CCD for spectral measurements, or through a series of tunable long- and short-pass filters for efficient wavelength selection, then to a single photon avalanche diode (SPAD) to perform photon counting and time-resolved fluorescence measurements via a time-tagging module.

For nonresonant excitation in PL and STED measurements, a green (532 nm) pulsed laser (∼50 ps pulse width) is used. In addition, a supercontinuum laser (SCL) fiber-coupled to an acousto-optic tunable filter (AOTF) provides a tunable pulsed excitation with a spectral range of 430 to 700 nm and a bandwidth of 1–2 nm. The excitation is pulsed, with a repetition rate of 78 MHz and a pulsewidth of a few ps. Depending on the spectral range selected this laser is used for both PLE and tunable STED. To stabilize the PL from the defect, a weak 450 nm blue CW-laser is also applied as in ref (29). For STED measurements the green laser is triggered by a voltage pulse from the SCL, which has a tunable delay, enabling control of the relative arrival time of excitation and depletion pulses.

In Figure 1c, the PL spectrum of defect-A in hBN is plotted in gray, along with PLE (blue) and STED (red) spectra. To a first approximation, it is clear that the PLE spectrum is the mirror image of the PL spectrum around the ZPL energy, where PL (PLE) probes the emission (absorption) spectrum. However, in a system such as hBN, the PL spectrum can be contaminated by light from other nearby emitters. Applying STED by scanning a red-detuned laser shows resonances at the PSB but eliminates stray emission from other emitters.

To perform STED, time-resolved PL is recorded from the ZPL of defect-A shown in Figure 1c. Following nonresonant excitation with a 532 nm PL-pulse, the emission decays exponentially with radiative lifetime of 3.58 ns, which is typical for hBN color centers. If the STED pulse, resonant with the PSB at ∼2.02 eV, arrives before the excitation pulse, the PL is not affected (see black trace in Figure 2b). However, if the STED pulse arrives after the excitation pulse, the PL is switched off on the time-scale of the laser pulse (red trace in Figure 2b). The STED pulse stimulates phonon-assisted emission, depleting the excited state, and suppressing the PL from the ZPL.

**Figure 2:**
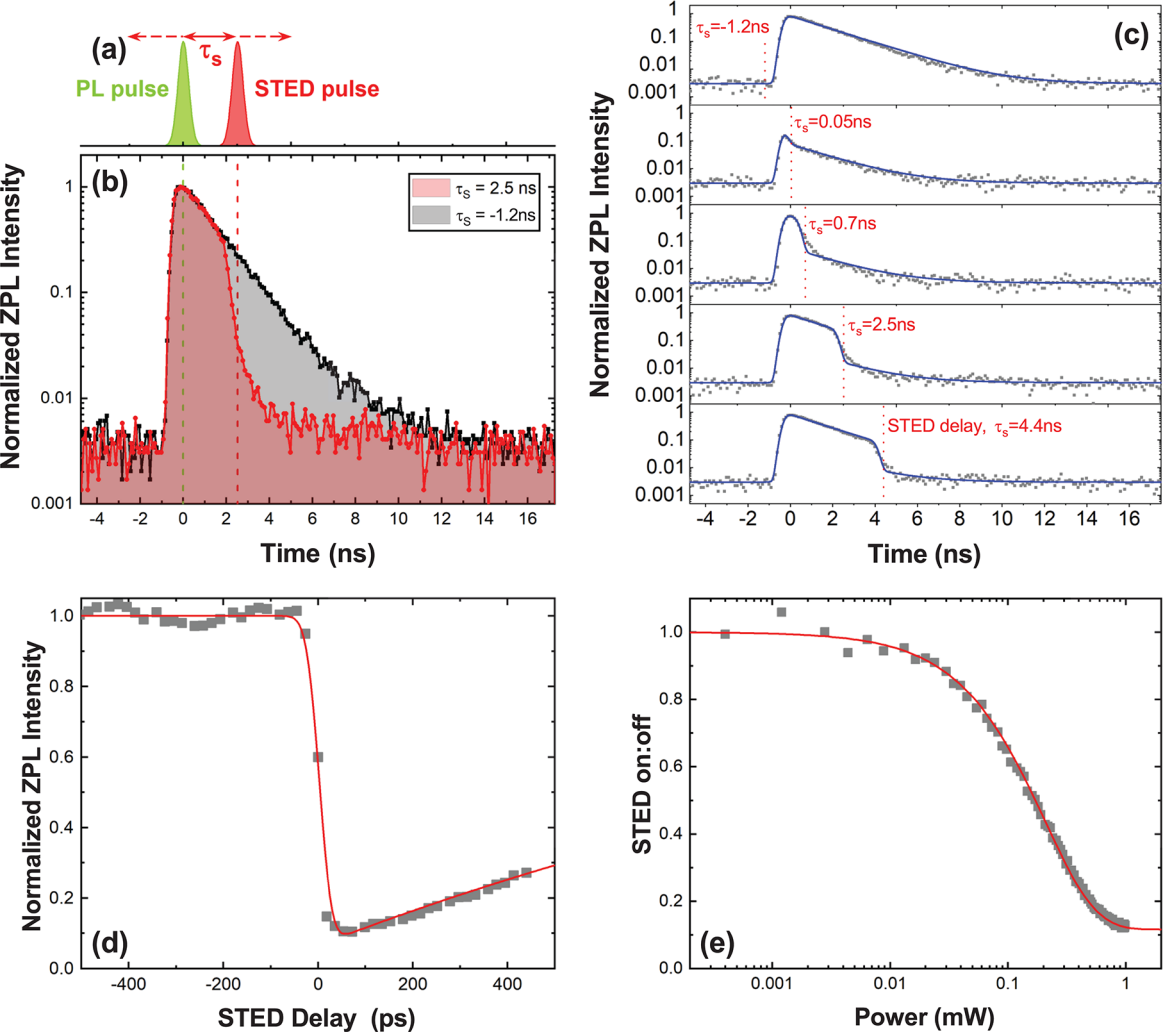
(a) Schematic of arrival times of excitation PL pulse and STED pulse, with delay τ_s_ between them. (b) Time-resolved ZPL-PL of defect-A with positive (red) and negative (black) τ_s_. The STED pulse switches off the emission. (c) Time-resolved PL of defect-A at five different values of τ_s_, showing rapid depletion at the arrival time of the STED pulse. (d) Plot of time-averaged ZPL-PL intensity with varying τ_s_. Moving the STED pulse through the PL pulse results in significant quenching of the PL signal that recovers as τ_s_ is increased. (e) Sweep of STED ratio with varying time-averaged power of the STED laser.

The gating of the ZPL PL by the STED pulse is further illustrated in Figure 2c, where time-resolved PL from defect-A are shown for five different values of τ_s_. In Figure 2d, the time-averaged PL is plotted against delay time. With negative τ_s_, the PL intensity is constant and sharply falls as the pulses overlap, recovering slowly as the STED pulse is moved through the radiative decay tail. This provides a method of performing STED measurements without varying the SCL laser energy or switching lasers on and off, which can affect the power of the lasers and thus the reliability of measurements: “STED on” pulses are set to arrive 100 ps after the excitation pulse, whereas “STED off” pulses arrive 100 ps before the excitation pulse. The STED on/off ratio is then recorded as the ratio between the STED on and off PL intensities. A power sweep was performed, varying SCL power (Figure 2e). Increasing SCL power decreases the STED ratio down to an apparent saturation at about 0.12. Experimental results were compared to simulations using a simple three-level rate equation model and show good agreement (see Supporting Information for details on the model used).

A comparison of STED, PL, and PLE spectra for defect-A, along with four other defects with similar ZPL energy is made in Figures 3 and 4. For comparison, the magnitude of the detuning from the ZPL is used, because as noted above, PLE uses a laser detuned to higher energies, whereas STED uses a laser detuned to lower energies. As reported previously,^30−33^ the shape of the PSB corresponds closely to the phonon dispersion relation for bulk hBN (Figure 3a).^27,34^ A detailed spectrum of the PL (emission) optical sideband (OPSB) along with corresponding PLE (absorption) data is shown in Figure 3b. The resolution of the PLE spectra is limited by the bandwidth of the SCL, and the PL shows sharper features. We note that the PL spectra has additional peaks not observed in PLE or STED. We attribute this to emission from other nearby emitters that are weakly excited (see Supporting Information for PLE spectra and polarization-resolved PL that support this attribution). Hence, we only compare the PLE and STED spectra. The absorption (emission) PSB maps the coupling of the excited (ground) state of the radiative transition to the single phonon vibronic states. For defect-A (Figure 3), two main peaks are observed in both PLE and STED. The peak corresponding directly to the maximum of the phonon density of states^32^ appears in both PLE and STED at ∼165 meV. In STED, probing the phonon sideband of the ground state, the peak at 200 meV matches the *LO*(*E*_1u_, Γ) mode energy in bulk hBN.^27^ In PLE, probing the phonon sideband of the excited state, the peak corresponding to the *LO*(*E*_1u_, Γ) phonon mode is red-shifted to 195 meV. Note that for *E*_1u_ (*E*_2g_) phonon modes, the adjacent planes of the crystal oscillate in- (out-of-) phase. Consequently, the in-plane electric field generated by the optical phonon modes interfere constructively (destructively).^34^ This implies that when the defect is in the excited state, the lattice is distorted along a lattice coordinate with *E*_1u_-like symmetry (in-plane dipole *x*,*y*), softening the spring-constant due to the anharmonicity of the bonds. We estimate this distortion to be about +2.6% of the lattice constant (see Supporting Information for calculation).

**Figure 3:**
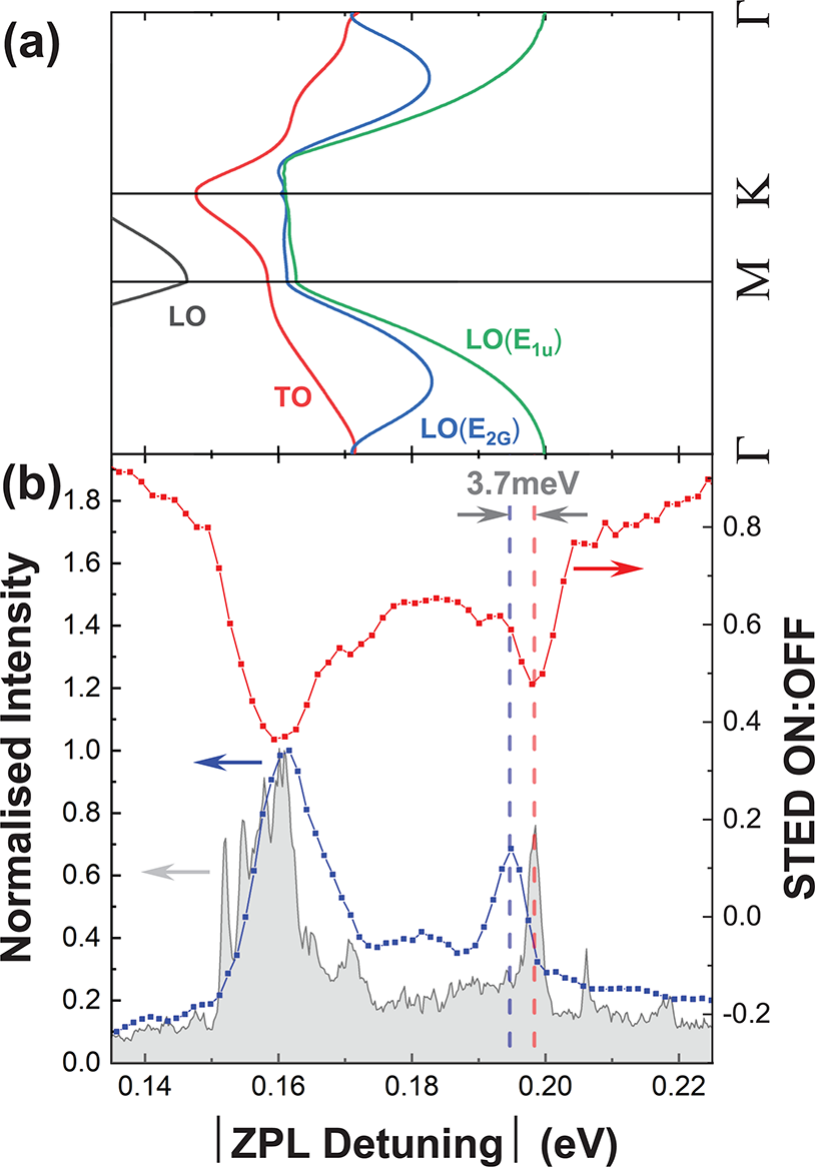
(a) Calculated optical phonon dispersion for bulk hBN with *LO*(*E*_1u_) mode highlighted. Taken from Serrano et al.^27^ (b) Comparison of PL (gray), PLE (blue), and STED (red) OPSB spectra of defect-A, which has a ZPL energy of 2.170 eV. Red (blue) dashed line shows position of the *LO*(*E*_1u_, Γ) transition in STED (PLE).

**Figure 4:**
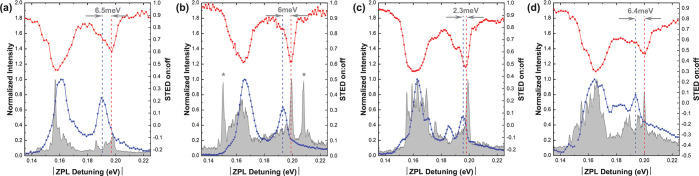
(a–d) Comparison of PL, PLE, and STED spectra from four similar defects with ZPL energies of (a) 2.166, (b) 2.175, (c) 2.142, and (d) 2.171 eV. Each defect shows shift in 200 meV peak between absorption and emission. In (b), the peaks marked with * are from another nearby defect or defects.

We now compare our spectroscopy results to candidate defects. Recently, Mendelson et al.^19^ have demonstrated that there is a carbon related defect emitting at 2.10 ± 0.04 eV with sharp ZPL, in-plane linear polarization, and nanosecond-scale radiative lifetimes, which matches our measurements. Of the possible carbon-related defects, the (1) → (1) transition of the *V*_*B*_*C*_*N*_^–^ line defect is the prime suspect. It has an in-plane linearly polarized optical dipole perpendicular to the axis of the defect. Our observations are consistent with this claim. In addition, in a previous work^29^ on a similar defect to defect-A, we observe a second ZPL peak in absorption orthogonally polarized to and ∼0.5 ± 0.1 eV above the emission ZPL, at 2.4–2.8 eV. If the defect is *V*_*B*_*C*_*N*_^–^ with *C*_2*v*_ symmetry, this should correspond to the (2) ↔ (1) transition, which is calculated in the supplement of ref (19) to have a vertical absorption energy between 2.8 and 3.6 eV. Since this state does not appear in emission and was observed in PLE of the ∼2 eV ZPL, this indicates a fast nonradiative relaxation to the (1) state that flips the polarization of the optical dipole, suggesting an intercrossing of the (2) and (1) states. In this picture, the ground-state is energetically isolated with no in-plane dipole component, and should have a weak lattice distortion compared to the excited state where there are number of orbital states with similar energy available for admixing.

Hayee et al.^35^ report three classes of defect emitters in the 2–2.2 eV energy range, which they suggest could be the *V*_*N*_*N*_*B*_, *V*_*N*_*C*_*B*_ or *V*_*N*_*O*_2*B*_ defects.^16^ All three share *C*_2*v*_ symmetry with the *V*_*B*_*C*_*N*_, so in principle all could exhibit similar behavior. We are unaware of any theoretical studies of selection rules for *V*_*N*_*O*_2*B*_, so no concrete statements can be made about it. For all charge states of *V*_*N*_*N*_*B*_(36) and *V*_*N*_*C*_*B*_(19,37) (and neutral and positive charge states of *V*_*C*_*B*_*N*_), transition energies of ∼2 eV are predicted to have out-of-plane optical dipoles, whereas the defects we study have in-plane optical dipoles.

Hayee et al.^35^ also discuss the *V*_*B*_ defect, and rule it out due to their sample preparation method, but we cannot. By contrast to the other candidate defects, the *V*_*B*_ point defect has *D*_3*h*_ symmetry, and the singly negatively charged *V*_*B*_^–^ is the only stable charge state to have in-band gap transitions.^38^ The ground state is calculated to have *A*_2_^′^ symmetry, and there exists an excited state with *E*′ symmetry^39^ variously calculated to emit at 2.287,^40^ 1.92,^41^ and 2.22 eV.^39^ As this state has a 2-fold degeneracy in the plane of the crystal, the Jahn–Teller effect occurs, deforming the defect and giving a preferred polarization direction. This deformation could also give rise to the shift in the *E*_1u_ phonon energy in the excited state. Reimers et al.^39^ also suggest there is an absorption resonance to the *E*′ state at 2.65–2.76 eV, which is consistent with the energy of the orthogonal absorption peak previously observed in ref (29). While *V*_*B*_^–^ has been proposed as responsible for emission around 2 eV,^15,41^ it has also been suggested that the *E*′ excited state undergoes rapid relaxation to a lower *E*″ energy state, resulting in emission at 1.76 eV.^39^ This peak has been observed in PL and ODMR measurements of hBN attributed to *V*_*B*_^–^.^14^ Hence, the likelihood of our observations being due to *V*_*B*_^–^ is unclear.

In this work, we have demonstrated the application of STED spectroscopy to the examination of vibronic states of defect emitters in hBN. We have shown that STED spectroscopy replicates the PL spectra, but with the advantage that STED completely eliminates stray signals from nearby emitters, making it immensely useful for systems with randomly placed emitters in the solid state, such as defects in 2D materials or self-assembled quantum dots. We have shown that STED is analogous and complementary to PLE spectroscopy, where the major difference is that STED probes vibronic spectra of the ground state in a two-level system, whereas PLE probes that of the excited state. We have then used STED and PLE to compare the vibronic spectra of the ground and excited states of the radiative transition. For color centers emitting near 2.2 eV, the main qualitative difference between the ground and excited states is a red-shift in the LO-phonon mode with *E*_1u_ symmetry. We compare our findings to recent work on different defect species in hBN and show that they are most consistent with the (1) → (1) transition in the *V*_*B*_*C*_*N*_^–^ defect. In this case, the shift to the phonon mode would be ascribed to a lattice distortion due to admixing between nearby excited states in the defect.

Here, the STED spectral resolution is limited to ∼1 nm by the AOTF filtering the SCL, which prevents a detailed examination of the OPSB around 165 meV. However, with a narrower linewidth laser, PLE/STED would enable further investigation of the fine structure of the OPSB and thus shed more light on electron–phonon coupling in hBN defects.

## Supporting Information Available

Details of theoretical modeling; estimation of lattice constant distortion between excited and ground state; PLE spectra and polarization-resolved PL of ZPL, PSB peaks, and extra peaks for defect in Figure 4b (PDF)
