# Supplementary material for: Stimulated Emission Depletion Spectroscopy of Color Centers in Hexagonal Boron Nitride
Source: ACS Photonics. 2021 Apr 7;8(4):1007–12. doi: 10.1021/acsphotonics.0c01917 (PMC8155571; doi:10.1021/acsphotonics.0c01917)
Supplement: Supplementary file 1 — ph0c01917_si_001.pdf [file ph0c01917_si_001.pdf]

Supporting Information: Stimulated emission depletion spectroscopy of color centers in hexagonal boron nitride

Ralph Nicholas Edward Malein,*,† Prince Khatri,† Andrew J. Ramsay,‡ and Isaac J. Luxmoore*,†

†*College of Engineering, Mathematics and Physical Sciences,University of Exeter, Exeter EX4 4QF, United Kingdom*

‡*Hitachi Cambridge Laboratory, Hitachi Europe Ltd., Cambridge CB3 0HE, United Kingdom*

E-mail: r.n.e.malein@exeter.ac.uk; i.j.luxmoore@exeter.ac.uk

List of Figures

S1 Level schematic of three-level system with rate parameters labeled. S-2

S2 **(a)**: STED (red) and PL (gray) PSB spectra of defect in Fig. 4(b). **(b)**: PLE spectra measured by filtering on energies of ZPL and PSB peaks in **(a)**. **(c)**: Polarisation-resolved PL (excitation at 532nm) of ZPL and PSB peaks in **(a)**. Colors of lines and markers correspond to PL peaks throughout. S-6

List of Tables

S1 Table of simulation parameters for 3-level model shown in Fig. S1. . . . . . . S-3

Model details

To verify that STED is responsible for the experimental observations, we compare the results to a simple three-level rate equation model based on the level schematic in Fig. S1. G and E are the ground and excited state populations of the radiative transition respectively; M is the intermediary vibronic level, lying above the ground state by the phonon mode energy.

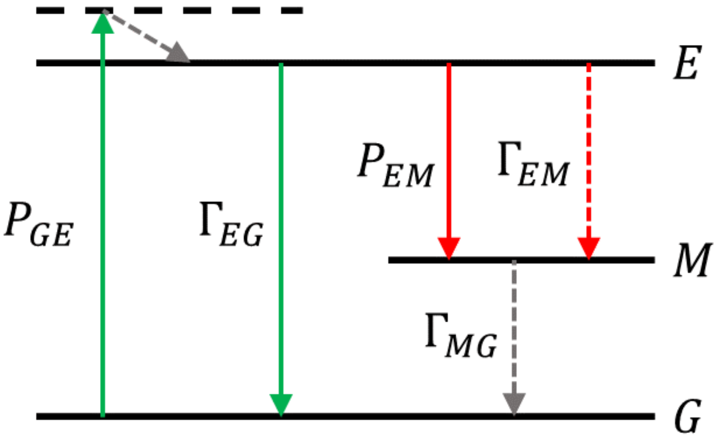

Figure S1: Level schematic of three-level system with rate parameters labeled.

The excitation pulse is modelled as a single pump rate encompassing excitation from G to a vibronic state above E followed by rapid relaxation to E (P_{GE}), whereas the STED pulse is a pump from E to M (P_{EM}). It is assumed that the non-radiative relaxation from M to G (Γ_{MG}) is fast compared to the other transitions. For time-resolved simulations, the excitation and STED pulses are modelled as Gaussian, and the simulated traces are then convolved with the IRF of the SPAD (Gaussian with 800 ps FWHM) to better fit to the measurement resolution. The ZPL intensity is then proportional to the population in E multiplied by the radiative decay rate Γ_{EG}. A phonon-mediated spontaneous emission rate Γ_{EM} is also included to model emission into the PSB. The rate equations are:

$$\dot{G} = \Gamma_{EG}E + \Gamma_{MG}M - P_{GE}G$$
$$\dot{E} = P_{GE}G - (\Gamma_{EG} + P_{EM} + \Gamma_{EM})\,E$$
$$\dot{M} = -(\dot{E} + \dot{G})$$

All simulations show good agreement with experimental data, with consistent model parameters (see Table S1): fitting to the time-resolved PL traces in Fig. 2(c) gives values of the radiative decays and allows accurate determination of the true pulse widths of the 532nm laser and SCL pulses; fitting the power sweep in Fig. 2(e) shows that the saturation is due to the short delay between excitation and STED pulses which allows a small degree of PL to be emitted before depletion of E.

Table S1: Table of simulation parameters for 3-level model shown in Fig. S1.

model parameter	value
Γ_{EG}, Γ_{EM}	279.3 MHz
Γ_{MG}	1 THz
P_{GE}	8 GHz
P_{EM} in delay sweep (Fig. 2(d))	1.29 THz
Excitation laser pulse width	50 ps
STED pulse width	2 ps

Estimation of lattice distortion

The distortion in the lattice is estimated by assuming a softening of the spring constant K of the phonon mode due to the distortion. For simplicity's sake, we consider a 1D diatomic chain whose dispersion is given by

$$\omega^2 = K\frac{m_B + m_N}{m_B m_N} \pm \sqrt{K^2\left(\frac{(m_B + m_N)^2}{m_B^2 m_N^2} - \frac{4}{m_B m_N}\sin^2\left(\frac{ka}{2}\right)\right)}$$

where k is the phonon momentum, a is the lattice parameter, and $m_{B,N}$ are the masses of the two atoms. As the interaction take place at the Γ point $k = 0$, the dispersion simplifies to

$$\omega^2 = K\frac{m_B + m_N}{m_B m_N} \pm \sqrt{K^2\frac{(m_B + m_N)^2}{m_B^2 m_N^2}}$$
$$= 0 \quad \text{or} \quad 2K\frac{m_B + m_N}{m_B m_N}$$

Thus in the optical branch, $\omega^2 \propto K$. While hBN is not a diatomic chain, considering units of K, this proportionality holds for a 2D hexagonal lattice.

Considering Hooke's law

$$K \propto \left.\frac{\partial^2 V}{\partial q^2}\right|_{q=q_0}$$

where V is the bond potential, q is the normal mode lattice coordinate and q_0 is the equilibrium lattice coordinate. Assuming a form of the potential similar to the Lennard-Jones potential, where it is minimized at the equilibrium position, and expanding around the equilibrium, we find that

$$\left.\frac{\partial^2 V}{\partial q^2}\right|_{q=q_0} \propto \frac{1}{q_0^2}$$

so

$$\omega \propto \frac{1}{q_0}$$

Thus, considering excited (ground) phonon mode energies $\hbar\omega_E = 195 \pm 2$meV ($\hbar\omega_G = 200$meV) and equilibrium positions q_E (q_G) we can calculate the excited state lattice equi-

librium position as a percentage of the ground state equilibrium:

$$\begin{aligned}\frac{q_E}{q_G} &= \frac{\hbar\omega_G}{\hbar\omega_E} \\ &= \frac{200}{195 \pm 2} \\ &= 102.6 \pm 1\%\end{aligned}$$

Leading to an overall distortion of $+2.6 \pm 1\%$.

PLE and polarisation-resolved PL of peaks in Fig. 4(b)

Fig. S2 shows spectroscopic data of the ZPL and PSB peaks for the defect in Fig. 4(b). In Fig. S2(a), the STED and PL spectra for the PSB show good agreement for the features at 0.165eV ($\mathrm{P_B}$) and 0.20eV ($\mathrm{P_C}$), but the PL shows two more narrow peaks at 0.15eV ($\mathrm{P_A}$) and 0.21ev ($\mathrm{P_D}$), suggesting that these are not due to the defect in question and are emitted by nearby defects or impurities. This is supported by Fig. S2(b) shows PLE spectra obtained by placing a tunable filter on the peaks indicated and sweeping the excitation laser through the PSB. It is clear that $\mathrm{P_B}$ and $\mathrm{P_C}$ show clear similarity to the PLE obtained form collecting the ZPL, whereas $\mathrm{P_A}$ and $\mathrm{P_D}$ show markedly different PLE spectra. Further evidence is given in Fig. S2(c), which shows polarisation-resolved PL for these peaks. While the gray (ZPL), blue ($\mathrm{P_B}$) and green ($\mathrm{P_C}$) curves show very similar polarisation, the red curve ($\mathrm{P_A}$) shows polarisation rotated by 30° counterclockwise compared to the ZPL, and the purple curve ($\mathrm{P_D}$) shows no strong polarisation at all. From this data we can conclude that $\mathrm{P_A}$ and $\mathrm{P_D}$, the peaks marked with asterisks in Fig. 4(b), are not due to phonon-mediated transitions from the defect being measured, but are due to stray light from another source.

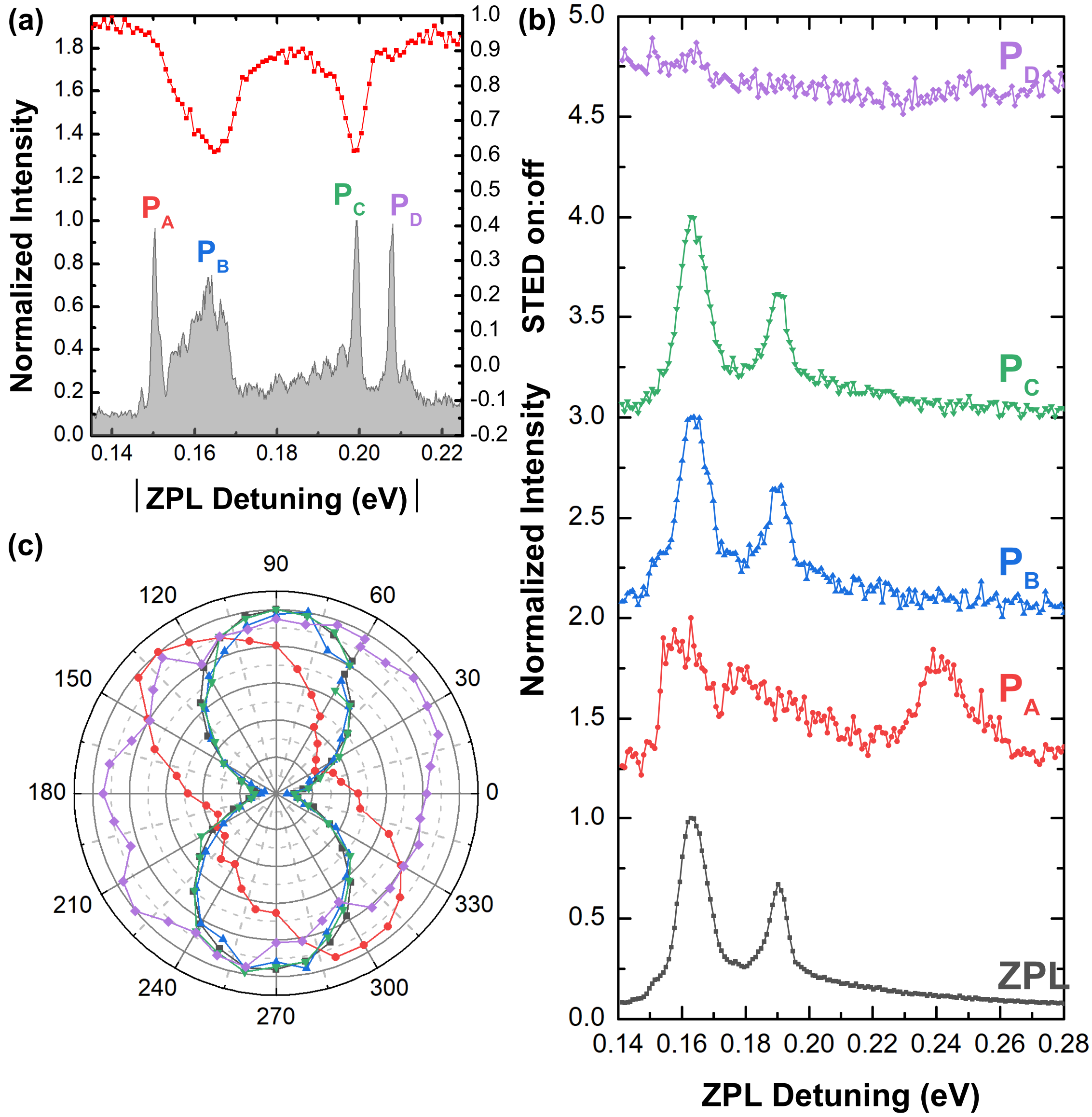

Figure S2: **(a)**: STED (red) and PL (gray) PSB spectra of defect in Fig. 4(b). **(b)**: PLE spectra measured by filtering on energies of ZPL and PSB peaks in **(a)**. **(c)**: Polarisation-resolved PL (excitation at 532nm) of ZPL and PSB peaks in **(a)**. Colors of lines and markers correspond to PL peaks throughout.
